# Case report: video-assisted thoracoscopic surgery for pulmonary arteriovenous malformation using near-infrared fluorescence with indocyanine green

**DOI:** 10.1186/s13019-023-02351-w

**Published:** 2023-10-27

**Authors:** Tianxiao Han, Jian Gao, Zhenfan Wang, Jian Zhou

**Affiliations:** Department of Thoracic Surgery, Thoracic Oncology Institute, Peking University People’s Hospital, Beijing, China

**Keywords:** Indocyanine Green, Near-infrared fluorescence, Pulmonary arteriovenous malformation

## Abstract

**Background:**

Pulmonary arteriovenous malformation (PAVM) is an abnormal communication between pulmonary vasculatures and has an unclear boundary with surrounding lung tissues. At present, surgeons can only determine its location by preoperative imaging and intraoperative palpation, despite its soft texture. Indocyanine green(ICG), a near-infrared fluorophore, has been demonstrated useful in the accurate identification of vascular tissue. Therefore, we explored its application in PAVM cases.

**Case presentation:**

We present two PAVM cases using near-infrared fluorescence (NIF) with 25 mg ICG at 5 mg/ml to achieve intraoperative visualization of the lesion in video-assisted thoracoscopic surgery (VATS). Under the NIF mode, ICG systemic injection led to successive signaling of the anomaly and normal tissues in merely 10 s, which helped us distinguish them efficiently and precisely. A peak signal-to-background ratio of 2.2 confirmed the significant fluorescence difference and excluded interference from carbon dust.

**Conclusions:**

We are the first to report the use of such an approach in delineating the margin of vascular malformation with high contrast, and this new finding may help minimize the damage to lung function in PAVM treatment. Further exploration and validation are needed to determine its role.

**Supplementary Information:**

The online version contains supplementary material available at 10.1186/s13019-023-02351-w.

## Background

Pulmonary arteriovenous malformation (PAVM) is characterized by an aberrant connection between a pulmonary feeding artery and the drainage vein, which bypasses the normal intervening capillary bed. It has been reported that over 80% of such cases are related to hereditary hemorrhagic telangiectasia [1]. The hallmark of this disease is significant extracardiac right-to-left blood shunt, leading to decreased oxygen saturation. Moreover, PAVM predisposes patients to potential complications such as severe hemoptysis, paradoxical embolism with brain abscess, and stroke [2]. Therefore, early detection and intervention are of great concern. Among these, video-assisted thoracoscopic surgery (VATS) has been increasingly performed due to its definitive efficacy and minimal injury.

However, surgeons often experience difficulties in locating small PAVMs and accurately discriminating them from normal tissue to completely remove lesions while minimizing damage to lung function. Traditionally, they can determine the boundary by either visual inspection or manual palpation. Challenges exist because such lesions are too soft to be distinguished by palpating. Moreover, deposits of carbon dust often interfere with their judgment. Therefore, the majority of previously reported PAVM cases treated by VATS are peripherally located [3–5]. Near-infrared fluorescence (NIF) imaging with indocyanine green (ICG) has recently emerged as a promising method to localize pulmonary nodules. A growing series of publications demonstrated the efficacy of ICG in vascular tissue identification [6–9]. Considering its wide usage in angiography, it might help guide the detection of pulmonary vascular malformations.

To date, this new strategy has not yet been implemented in PAVM patients. Here, we report a new clinical attempt worldwide using intraoperative NIF imaging with ICG to visualize the margins of lesions in two PAVM patients.

## Case presentation

**Fig. 1:**
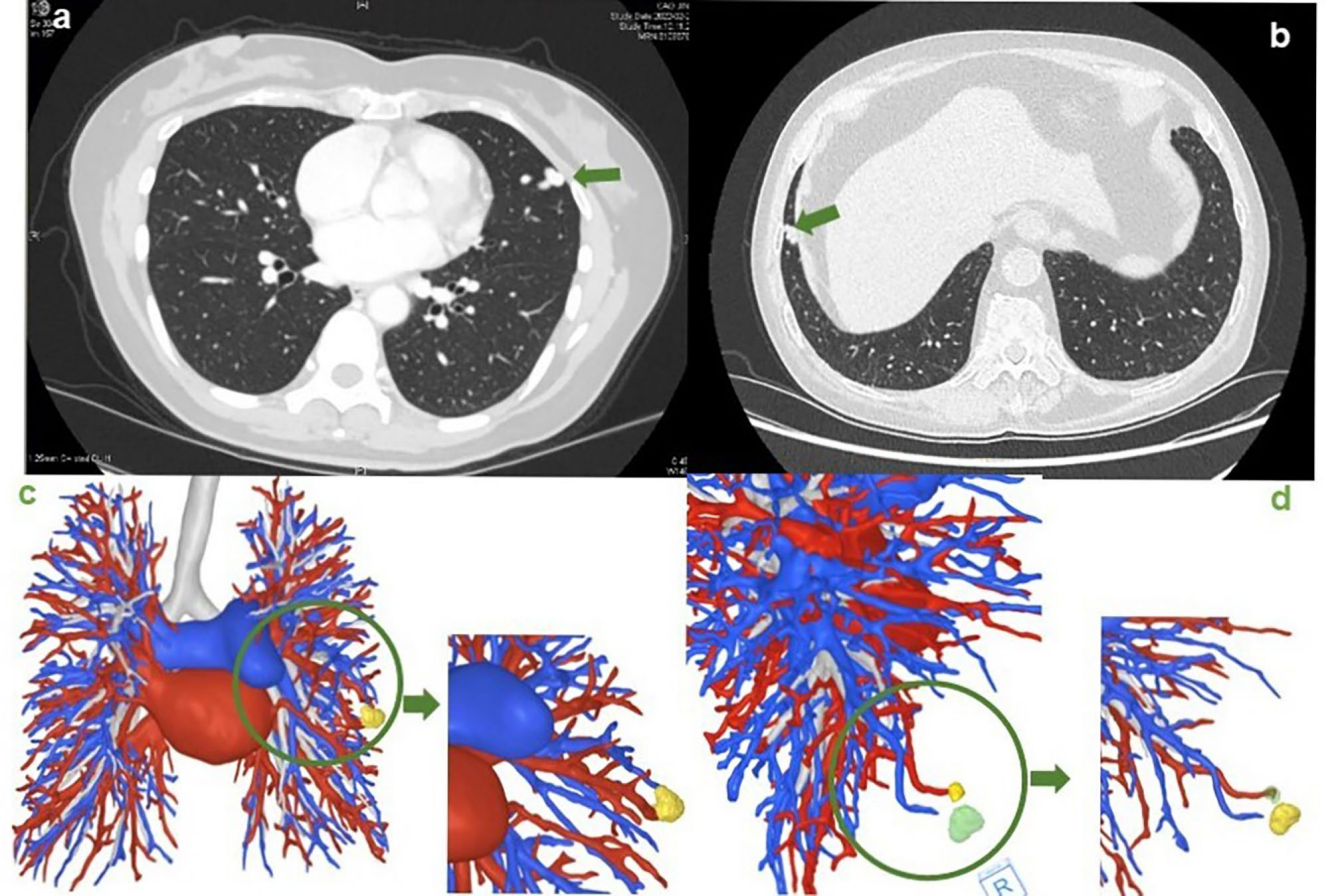
CT and 3-dimensional imaging for two cases. **a**. CT view of the first patient shows a nodular opacity in the lingual segment of the left upper lobe; **b**. CT view of the second patient shows a nodule in the anterior basal segment of the right lower lobe **c**. 3-dimensional imaging of the first patient’s lung vasculature; **d**. 3-dimensional imaging of the second patient’s lung vasculature

The first 52-year-old woman, who revealed exertional dyspnea and occasional chest pain for four months, came to us for surgical treatment. She reported no symptoms at rest and no hemoptysis. Her arterial blood gas analysis at admission showed SaO_2_ of 92.1% and PO_2_ as low as 66.8 mmHg. A computed tomography (CT) thorax with contrast scan revealed a low-density nodular opacity in the lingual segment of the left upper lobe measuring 8 mm. The lesion was demonstrated as a PAVM on a contrast-enhanced scan, presenting with the feeding lingual artery and the twisted draining vein **(**Fig. 1a**)**. Percutaneous transcatheter embolization (PTE) was not adopted due to the high risk of recanalization. Furthermore, such a peripherally located solitary nodule was an excellent candidate for VATS resection. The second patient was a 72-year-old man. His checkup enhanced CT scan detected both a vascular malformation and a striped high-density shadow in the right lung. Due to increased FDG uptake in positron emission tomography-CT analysis, the nodule was further determined as cancer, which indicated a confined operation. Similarly, the thoracic CT imaging showed a tortuous vascular mass at the pleura of the anterior basal segment of the right lower lobe formed by an aberrant artery and a branch of the vein **(**Fig. 1b**)**. After contraindication assessment and multidisciplinary discussion, we decided to perform thoracoscopic wedge resection for the PAVM under the aid of near-infrared (NIR) fluorescence imaging with ICG during the confined operation for the tumor. For both patients, the reconstruction of PAVMs by preoperative 3-dimensional CT scan could be a reference **(**Fig. 1c and d**)**.

The two patients were anesthetized with double-lumen endotracheal intubation, and operations were completed on constant single-lung ventilation. Two trocar ports were used to enter the thoracic cavity. The entire procedure was performed using a D-light P® NIR thoracoscope system (KARL STORZ GmbH & Co, Germany) through the observation port.

**Fig. 2:**
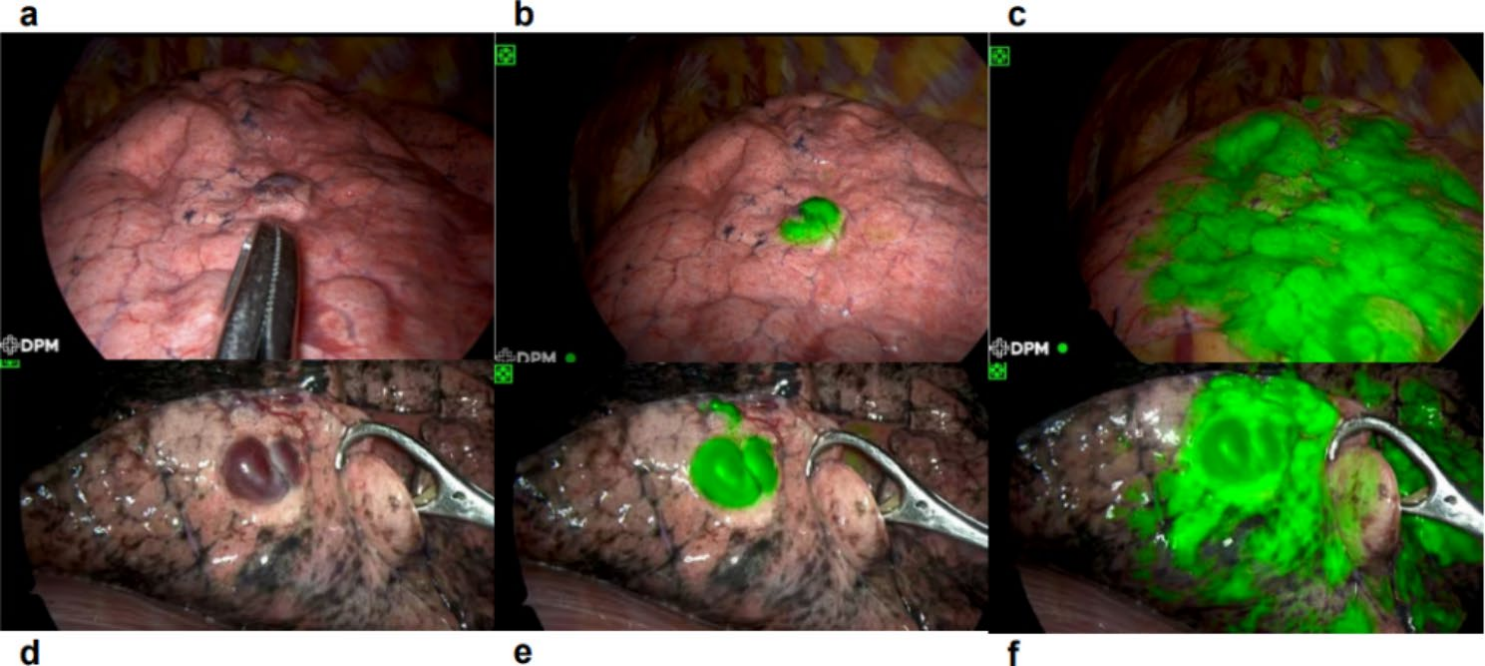
Intraoperative view under conventional mode and NIF mode. **a**. Intraoperative findings of the first patient under conventional mode; **b**. The earliest developed tissue of the first patient under NIF mode; **c**. Spreading fluorescence signal over time under NIF light in the first patient; **d**. Intraoperative findings of the second patient under conventional mode; **e**. The earliest developed tissue of the second patient under NIF mode; **f**. Spreading fluorescence signal over time under NIF light in the second patient

For the 52-year-old woman, a sac-like lesion (green arrow) could be seen at the surface of the lingual segment of the left upper lobe under normal thoracoscopic mode (Fig. 2a), whereas the boundary was unclear. Despite extensive manual palpation, the lesion remained unclear. To minimize the impact on respiratory function, we chose NIR fluorescence with ICG for further inspection. We rapidly injected 25 mg ICG (Dandong Yichuang Pharmaceutical Co. Ltd., China) at 5 mg/ml into her left radial vein, and the surgical field was viewed under fluorescence mode. The targeted PAVM began to display a prominent fluorescence signal five seconds after ICG injection and earlier than the surrounding area, permitting distinction of the lesion from the normal lung (Fig. 2b, Additional File 1). The perfused ICG allowed the remainder of the lung to become fluorescent green five seconds afterward (Fig. 2c). It took approximately 20 s for the whole surgical area to develop. We encountered a similar occasion in operation for the second man (Fig. 2d, Additional File 2). Under the NIR light, there was also a circumscribed area that fluoresced preceding other parts (Fig. 2e and f) and was consistent with the preoperative CT scan. Therefore, we could ascertain the location and determine the resection line. Parenchymal wedge resections were then performed just outside the boundary using surgical staplers for two patients. Subsequently, no air leak from the anastomosis was detected by inflation of the lung under water. As for the second patient’s cancerous nodule in the upper lobe, we first performed a wedge resection. We continued to perform a lobectomy after the intraoperative frozen section revealed its malignant nature.

**Fig. 3:**
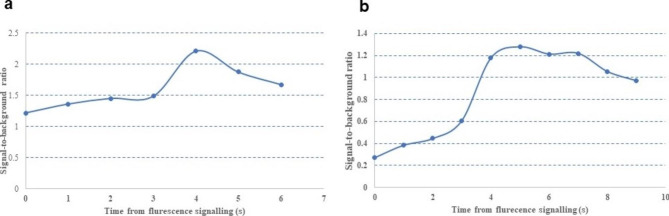
Signal-to-background ratio curves for two cases

Signal-to-background ratios (SBR) over time were calculated using ImageJ digital analysis software for them, the maximum of which was 2.2 for the woman **(**Fig. 3a**)** and 1.3 for the man **(**Fig. 3b**)**.

After the operation, the arterial blood gas analyses found oxygenation greatly improved; the SaO_2_ was 99.9%, PO_2_ was 295.3 mmHg without oxygen inhalation for the woman, and FiO_2_ elevated to 462.2 mmHg for the man. Their postoperative pathological findings confirmed dilated vascular malformations. Ultimately, both patients were discharged after uneventful postoperative courses.

## Discussion

Here we report new clinical cases using NIF imaging with systemically injected ICG to achieve thoracoscopic sublobar resection of PAVMs.

As a rarely occurring disease, most cases of PAVM are congenital. According to previous retrospective studies, at least one-third of such patients had multiple lesions. Up to 80% were peripherally located and closely related to the pleura [10]. Treatment is indicated for asymptomatic patients with feeding arteries exceeding 3 mm in diameter due to their significant risk of suffering from neurologic complications or symptomatic patients with lesions regardless of size [11].

Historically, lobectomy was preferred to ensure the complete removal of abnormal tissue [12]. Given the severely impacted respiratory function, it was gradually replaced by PTE, which became a first-line treatment with advances in interventional techniques. Despite being less invasive, it bears the risk of recanalization and accidental systemic embolism. According to a systematic review of the Cochrane database, no randomized controlled trials (RCTs) have proven PTE to be the mainstream treatment for PAVMs [13]. Moreover, some vascular mimickers of PAVMs require surgical resection [14]. The advent of thoracoscopy has unleashed the potential for radical and least-invasive treatment. Sublobar lung resection by VATS has gradually become the potentially optimal choice, especially for those patients with solitary or peripheral clustered lesions. To date, only a few successful cases of wedge resection have been reported [3, 4, 15–17]. Shiiya H and his team [18] described a solitary PAVM located adjacent to the visceral pleura and removed under VATS wedge resection with the help of MDCT and 3DCT. For most reported cases of PAVMs, the feeding artery, draining vein, and aneurysmal lesion were surgically divided to completely resect, which seems difficult for small lesions compared with larger ones [19].

For wedge resection, precise excision is desirable, because under-resection may cause recurrent diseases, and over-resection decreases respiratory capacity. Discerning the border between the normal lung and the PAVM is crucial during the operation. Given the difficulties of visual inspection confused by carbon dust and manual palpation thwarted by soft texture, it is difficult to achieve this goal. The palpation of the finger may not discriminate small and soft nodules and is more limited in thoracoscopic surgeries. In our experience, vascular lesions are quite soft and those amenable to wedge resection are usually small. This may lead to fuzzy recognition. In the study conducted by Okusanya OT [20], patients with nodules to resect were given systemic ICG and then underwent an open thoracotomy. The NIR imaging can detect pulmonary nodules that are difficult to discriminate on finger palpation. Our cases reported marginally distributed PAVMs suitable for wedge resection. To achieve precise resection, we tried intravenous injection of ICG to ascertain the location and border of anomalies. Notably, intravenous injection of ICG for PAVM surgical treatment is reported here for the first time.

ICG, as a rapidly cleared fluorescence contrast, has been approved by the U.S. Food and Drug Administration as an intravenously injected drug [12]. Real-time NIR fluorescence imaging with systemic ICG is a novel modality used in thoracic surgery to help identify lung nodules and demarcate the intersegmental plane in lung segmentectomy [21, 22]. ICG is a non-specific fluorescein-like dye to help delineate the margins of vascular anatomy under direct visualization. It can be used to distinguish AVM vessels from normal vessels based on the timing of fluorescence with the dye. Previously, it has been used successfully in ophthalmic angiography, cerebral perfusion assessment, and cerebral arteriovenous malformations [23]. We were therefore inspired to apply it to identify PAVMs and abnormal blood flows in the lung.

ICG binds primarily to globulins and remains within the vessels upon rejection. It is cleared exclusively by the liver and is neither metabolized nor reabsorbed from the intestine. The infrared light is directed to the surface of the lung from the light source through a special filter, and the reflected light by the contrast is detected by a camera. The signals form images according to the setup transducer on the monitor. If the tissue receives cardiac output, its appearance is affected by ICG. Therefore, this method can delineate the lesion boundary according to the degree of blood flow. Once injected into the vein, ICG returns to the right heart through systematic circulation and is immediately pumped into the pulmonary artery. Without passing through the pulmonary circulation into the left heart and the peripheral arteries, ICG can fill and outline the PAVM. In the overlay imaging window, the normal tissues show uniformly fluorescent green, while the lesion will be depicted as the first station to be filled. Consequently, NIR with ICG rapidly differentiates the PAVM from the surrounding lung tissue by their relative filling time.

In our patients, fluorescence signals of the lesion and normal tissue were not detected simultaneously at the beginning. The video demonstrated that the PAVMs were first visualized approximately 10 s after ICG injection. The surrounding lung tissue began to show obvious signals 20 s later through blood circulation. The imaging distinction was apparent in both two patients. The dynamic SBR curves confirmed that NIR ICG imaging has a high resolution, consistent with the video. Although the peak value was lower in the second case due to his severe carbon dust deposition, both the two dynamic SBR curves increased to a crest followed by decreasing trend, thus exhibiting the outstanding potential of ICG under NIF imaging in locating vascular anomalies during lung resection surgery. Consistent with previous studies, the contrast between the nodules and the surrounding tissues was less obvious at lower SBR. Furthermore, the fluorescent signal was considered detectable with SBR ≥ 1.5 [24, 25]. Therefore, the intraoperative real-time NIF imaging system successfully navigated surgeons to find the PAVM. Moreover, no adverse reactions or allergic reactions occurred in our cases.

It is worth mentioning that failure to completely resect the vascular lesion may be one of the reasons for postoperative recurrence. With the aid of ICG-based NIR fluorescence imaging, surgeons can precisely resect such lesions and prevent the recurrence of pneumothorax. By removing malformed vascular structures completely, the risk of recanalization, collateralization, and peri-interventional paradoxical embolism may be reduced.

In most cases, the diagnosis is established after pathohistological examinations. Previous studies have pointed out that a multitude of vascular and nonvascular pulmonary lesions can mimic the appearance of PAVMs including pulmonary vein varix, fibrosing mediastinitis, lung granulomas, bronchoceles, ground-glass opacities, and atelectasis [26]. Identifying the vascular connections will allow distinguishing of PAVMs from their mimics. In many cases of neurological AVM [6–8, 27], the superficial feeders, drainers, and nidus can be identified easily with the aid of ICG. These experiences proved that microscope-integrated ICG angiography is a useful tool in AVM surgery. Based on these studies, we pioneered the utility of ICG in fluorescent thoracoscopic surgery to distinguish pulmonary AVM.

## Conclusions

In conclusion, we showed in our cases that systemic ICG injection was helpful in PAVM surgical treatment, from the perspective of accurate demarcation of the resection boundary. Moreover, NIF imaging may be helpful in cases of peripheral PAVMs that are difficult to identify using conventional methods. This is the first published report of NIR fluorescence with ICG in the sublobar resection of PAVMs. Considering the low risk for adverse side effects, ICG is beneficial in PAVM surgical treatment.

### Electronic supplementary material

Below is the link to the electronic supplementary material.

Additional File Video 1: The surgical video clip of the 52-year-old woman

Additional File Video 2: The surgical video clip of the 72-year-old man

Additional File Video 3: The preoperative CT imaging of the 52-year-old woman

Additional File Video 4: The preoperative CT imaging of the 72-year-old man

## Data Availability

Data sharing is not applicable to this article as no datasets were generated or analyzed during the current study.

## List of abbreviations

PAVM: Pulmonary arteriovenous malformation
ICG: Indocyanine green
NIR: Near-infrared
NIF: Near-infrared fluorescence
VATS: Video-assisted thoracoscopic surgery
CT: Computed tomography
PTE: Percutaneous transcatheter embolization
SBR: Signal-to-background ratio
RCT: Randomized controlled trial

## References

[1] Wong HH, Chan RP, Klatt R, Faughnan ME (2011). Idiopathic pulmonary arteriovenous malformations: clinical and imaging characteristics. Eur Respir J.

[2] Cartin-Ceba R, Swanson KL, Krowka MJ (2013). Pulmonary arteriovenous malformations. Chest.

[3] Akiyama S, Hanada S, Uruga H, Takaya H, Miyamoto A, Kishi K (2013). Hereditary hemorrhagic telangiectasia with pulmonary arteriovenous malformations and embolic strokes treated successfully with video-assisted thoracoscopic resection. Intern Med.

[4] Reichert M, Kerber S, Alkoudmani I, Bodner J (2016). Management of a solitary pulmonary arteriovenous malformation by video-assisted thoracoscopic surgery and anatomic lingula resection: video and review. Surg Endosc.

[5] Shiiya H, Suzuki Y, Yamazaki S, Kaga K (2018). Polypoid pulmonary arteriovenous malformation causing hemothorax treated with thoracoscopic wedge resection. Surg Case Rep.

[6] Takagi Y, Kikuta K, Nozaki K, Sawamura K, Hashimoto N (2007). Detection of a residual nidus by surgical microscope-integrated intraoperative near-infrared indocyanine green videoangiography in a child with a cerebral arteriovenous malformation. J Neurosurg.

[7] Killory BD, Nakaji P, Gonzales LF, Ponce FA, Wait SD, Spetzler RF (2009). Prospective evaluation of surgical microscope-integrated intraoperative near-infrared indocyanine green angiography during cerebral arteriovenous malformation surgery. Neurosurgery.

[8] Walsh DC, Zebian B, Tolias CM, Gullan RW (2014). Intraoperative indocyanine green video-angiography as an aid to the microsurgical treatment of spinal vascular malformations. Brit J Neurosurg.

[9] Kurata Y, Hayano K, Matsusaka K, Mamiya H, Uesato M, Matsubara H (2022). A case report of duodenal arteriovenous malformation: usefulness of intraoperative indocyanine green angiography for precise identification of the lesion. Surg Case Rep.

[10] BOSHER LH, BLAKE DA., & BYRD BR (1959). An analysis of the pathologic anatomy of pulmonary arteriovenous aneurysms with particular reference to the applicability of local excision. Surgery.

[11] Shovlin CL, Jackson JE, Bamford KB, Jenkins IH, Benjamin AR, Kulinskaya E (2008). Primary determinants of ischaemic stroke/brain abscess risks are independent of the severity of pulmonary arteriovenous malformations in hereditary hemorrhagic telangiectasia. Thorax.

[12] Georghiou GP, Berman M, Vidne BA (2003). Pulmonary arteriovenous malformation treated by lobectomy. Eur J Cardiothorac Surg.

[13] Hsu CC, Kwan GN, Evans-Barns H, van Driel ML (2018). Embolization for pulmonary arteriovenous malformation. Cochrane Database Syst Rev.

[14] Lee HN, Hyun D (2022). Pulmonary arteriovenous malformation and its vascular mimickers. Korean J Radiol.

[15] Iida T, Sato M, Nakajima J (2021). Resection of clustered arteriovenous malformations to avoid lung transplantation. Ann Thorac Surg.

[16] Sano A, Tsuchiya T (2015). Thoracoscopic surgery for multiple peripheral pulmonary arteriovenous fistulas. Ann Thorac Surg.

[17] Bakhos CT, Wang SC, Rosen JM (2016). Contemporary role of minimally invasive thoracic surgery in the management of pulmonary arteriovenous malformations: report of two cases and review of the literature. J Thorac Dis.

[18] Shiiya H, Suzuki Y, Yamazaki S, Kaga K (2018). Polypoid pulmonary arteriovenous malformation causing hemothorax treated with thoracoscopic wedge resection. Surg Case Rep.

[19] Temes RT, Paramsothy P, Endara SA, Wernly JA (1998). Resection of a solitary pulmonary arteriovenous malformation by video-assisted thoracic surgery. J Thorac Cardiovasc Surg.

[20] Okusanya OT, Holt D, Heitjan D, Deshpande C, Venegas O, Singhal S (2014). Intraoperative near-infrared imaging can identify pulmonary nodules. Ann Thorac Surg.

[21] Okusanya OT, Holt D, Heitjan D, Deshpande C, Venegas O, Jiang J (2014). Intraoperative near infrared imaging can identify pulmonary nodules. Ann Thorac Surg.

[22] Tarumi S, Misaki N, Kasai Y, Chang SS, Go T, Yokomise H (2014). Clinical trial of video-assisted thoracoscopic segmentectomy using infrared thoracoscopy with indocyanine green. Eur J Cardiothorac Surg.

[23] Takagi Y, Sawamura K, Hashimoto N, Miyamoto S (2012). Evaluation of serial intraoperative surgical microscope-integrated intraoperative near-infrared indocyanine green video angiography in patients with cerebral arteriovenous malformations. Neurosurgery.

[24] Chiti LE, Husi B, Park B, Beer P, D’Orchymont F, Nolff MC (2023). Performance of two clinical fluorescence imaging systems with different targeted and non-targeted near-infrared fluorophores: a cadaveric explorative study. Front Vet Sci.

[25] Kanniyappan U, Wang B, Yang C, Ghassemi P, Litorja M, Suresh N (2020). Performance test methods for near-infrared fluorescence imaging. Med Phys.

[26] Raptis DA, Short R, Robb C, Marlow J, Naeem M, Bhalla S (2022). CT appearance of pulmonary arteriovenous malformations and mimics. Radiographics.

[27] Ono H, Kusano M, Kawamata F, Danjo Y, Kawakami M, Nishihara H (2016). Intraoperative localization of arteriovenous malformation of a jejunum with combined use of angiographic methods and indocyanine green injection: report of a new technique. Int J Surg Case Rep.

